# Detection of Sulfur Mustard Adducts in Eye Compartments Following Ocular Exposure in Rabbits

**DOI:** 10.3390/metabo16100792

**Published:** 2026-10-19

**Authors:** Fanny Gros-Désormeaux, Fanny Caffin, Alexandre Igert, Jade Boudard, Sylvain Caillat, Jihéne Bouhlel, François Fenaille, Thierry Douki

**Affiliations:** 1 Institut de Recherche Biomédicale des Armées, 91223 Brétigny-sur-Orge, France; fanny.gros-desormeaux@def.gouv.fr (F.G.D.); fanny.caffin@def.gouv.fr (F.C.); alexandre.igert@def.gouv.fr (A.I.); 2 Univ. Grenoble Alpes, CEA, CNRS, IRIG, SyMMES UMR 5819, 38000 Grenoble, France; jadeboudard38@gmail.com (J.B.); sylvain.caillat@cea.fr (S.C.); 3 Département Médicaments et Technologies pour la Santé, Université Paris Saclay, CEA, INRAE, SPI, MetaboHUB IDF, 91191 Gif sur Yvette, France; jihene.bouhlel@inserm.fr (J.B.); francois.fenaille@cea.fr (F.F.)

**Keywords:** warfare agents, sulfur mustard, ocular toxicity, biochemical biomarkers, mass spectrometry

## Abstract

**Highlights:**

**What are the main findings?**

- Sulfur mustard adducts to cysteine and guanine are relevant exposure biomarkers in the eyes.
- Sulfur mustard adducts are detected in tears and in aqueous and vitreous humors but not in plasma or distant organs.

**What are the implications of the main findings?**

- A decreasing concentration gradient from tears to vitreous humor reflects the rapid reactivity of sulfur mustard upon initial contact with the ocular surface.
- The absence of detectable sulfur mustard adducts in blood and organs provides no evidence of systemic diffusion beyond the ocular sphere.

**Abstract:**

**Background/Objectives**: Sulfur mustard (SM) is an old warfare agent that remains a threat to both military personnel and civilians. SM contamination occurs through three main routes: dermal contact, inhalation, and ocular exposure. The clinical consequences of ocular exposure range from conjunctivitis and ulcerations to blindness. While these pathophysiological consequences are well-established, the mechanisms of action of SM in the eyes are not fully understood. **Methods**: The present study, which was conducted in rabbits, aimed at quantifying a series of biomarkers resulting from the electrophilic addition of SM to glutathione and nucleic acids in multiple biological matrices. This allowed us to investigate the distribution of SM within the ocular sphere and its diffusion to distant organs. **Results**: SM biomarkers were detected in large amounts in tears and aqueous humor but at substantially lower levels in the vitreous humor. No evidence of diffusion was observed from the exposed eye to plasma and distant organs, including the brain, liver, and lungs. **Conclusions**: These results are in agreement with a role for the cornea as a major sink for SM, thereby preventing damage to other ocular compartments and systemic diffusion.

## 1. Introduction

Sulfur mustard (SM, bis-(2-chloroethyl)-sulfide) is a chemical warfare agent first used during World War I. Since then, SM has been deployed in several conflicts, including recently in Iran and Syria [1,2]. Its ease of synthesis and the persistence of existing stockpiles also raise concerns regarding the potential use of SM in terrorist actions [3]. Another concern regarding SM is the occurrence of accidents with old or abandoned ammunitions [4]. Injuries to the skin and the respiratory track are two well-documented effects of exposure to SM [5,6,7], unlike ocular effects, which have been studied less extensively, even though the eye is the most sensitive organ. Ocular symptoms typically appear first after a latency period of 2 to 12 h. The initial involvement manifests as a foreign body sensation, intense lacrimation, photophobia, and blepharospasm. Clinically, severe conjunctivitis and palpebral edema are observed. At higher doses, keratitis (corneal involvement) may occur, ranging from simple epithelial erosion to complete opacification, which can lead to temporary or permanent blindness in the most severe cases, as well as persistent ulcerations [8].

These effects are explained by the persistence of pro-inflammatory mediators in the aqueous humor and the structural alteration of the basement membrane. These phenomena generate a deleterious microenvironment that promotes the development of a recurrent triad of lesions. Corneal neovascularization, resulting from the loss of the cornea’s angiogenic privilege, accompanies progressive corneal opacity linked to the disorganization of the stromal matrix. Concurrently, chronic epithelial instability induces persistent and recurrent corneal ulcers; their resistance to physiological repair mechanisms exposes the organ to major complications, such as keratomalacia or perforation, ultimately leading to permanent blindness [8,9,10,11,12]. Due to the absence of an antidote, medical management is limited to symptomatic treatments, which mostly rely on early irrigation and the instillation of antiseptic, healing, or anti-inflammatory eye drops to relieve pain [13,14,15]. In summary, the pathophysiological effects of sulfur mustard on the eye are relatively well-characterized, but the molecular and biochemical mechanisms underlying its ocular toxicity remain poorly understood and warrant further investigation.

The marked toxicity of SM can be explained by its chemical structure, which exhibits two chlorine atoms conferring high reactivity with biomolecules. Those include, among others, proteins [16,17,18,19], nucleic acids [7], and glutathione [16,20,21,22]. The resulting formation of adducts strongly impairs the cellular machinery, which leads to cell death or perturbation of physiological functions. The biological impact of SM has been demonstrated by proteomic [23,24,25,26] and metabolomic studies [27,28]. Formation and accumulation of these modified biomolecules represent opportunities for the development of specific molecular biomarkers [16,29,30]. Those can be used to determine the amounts of internalized SM following exposure. Determination of the concentration of biomarkers in specific organs can also provide insight into the physiological impact of SM.

In this field, our group has developed several assays combining liquid chromatography separation and mass spectrometry detection for the accurate quantification of biomarkers of exposure to SM and its 2-chloroethyl-ethyl-sulfide analog (CEES) in rodent models. Using these tools, we obtained deeper insight into the genotoxicity of SM through the measurement of DNA adduct levels in organs following dermal exposure [25,26,27,28]. Quantification in biological fluids, such as plasma and urine, of glutathione metabolites from the mercapturic acid pathway (e.g., adducts with NAC-SM, Cys-SM) and of the free guanine adduct (Gua-SM), resulting from depurination of nucleic acids, provided additional information [29,30]. In the present study, we used these tools to evaluate the impact of ocular SM exposure in rabbits, an animal known to be a relevant model of the human eye. Quantification of biomarkers (conjugates to glutathione and their metabolites, DNA adducts) was performed in tears, in several compartments of the eye, and in plasma and DNA isolated from several internal organs.

## 2. Materials and Methods

### 2.1. Chemicals

Formic acid (LC–MS grade) was purchased from Thermo Fisher Scientific (Rockford, IL, USA), acetonitrile (HPLC-MS grade) from Carlo Erba (Val de Reuil, France), and methanol (HPLC grade) and ammonium formate from Sigma Aldrich (Saint Quentin Falavier, France). Ribonuclease T1, ribonuclease A, phosphodiesterase II, deoxyribonuclease II, alkaline phosphatase, and nuclease P1 were obtained from Sigma. Protease was purchased from Qiagen and phosphodiesterase I from Worthington (Lakewood, NJ, USA). SM was supplied by the French “Direction Générale de l’Armement”. The standards of the targeted compounds, 2-hydroxyethyl-thioethyl-*S*-cysteine (Cys-SM), 2-hydroxyethyl-thioethyl-*S*-(*N*-acetylcysteine) (NAC-SM), and 2-hydroxyethyl-thioethyl-N7-guanine (Gua-SM), were prepared through incubation of cysteine, *N*-acetylcysteine, and 2′-deoxyguanosine, respectively, with 2-chloroethyl-2-hydroxyethyl-sulfide (ChemSpace, Riga, Latvia), as previously described [27]. The guanine adduct was obtained via the thermal depurination of the 2′-deoxyguanosine derivative. Isotopically labeled internal standards were synthesized using the same protocols with either [^13^C_3_,^15^N]-cysteine, [^13^C_3_,1^5^N]-*N*-acetylcysteine, or [^15^N_5_]-2′-deoxyguanosine.

### 2.2. Experimental Design

#### 2.2.1. Animals

This work was performed in accordance with the Directives of the European Communities Council Directive 2010/63/UE and was submitted to and approved by the ethical committee of the French Service de Santé des Armées (CEA-SSA) (approval code: n°500916/ARM/IGSSA/SP; approval date: 22 January 2020). Twenty-four 10-week-old female New Zealand white rabbits weighing 2.5–3.0 kg were received and housed individually. The principles of laboratory animal care were followed in all experiments. The animals were monitored daily to ensure their well-being, and they were provided a standard diet and water *ad libitum*. An environmental light cycle was set up with 12 h of light and 12 h of darkness per day, with a maximum light intensity of 40 lux to reduce photophobia. Rabbits were subjected to 1 week of quarantine followed by 2 weeks of habituation before SM exposure.

#### 2.2.2. SM Exposure

Exposures were performed in the toxicology laboratories of the French “Institut de Recherche Biomédical des Armées”. Handling precautions related to SM included the use of personal protective equipment and handling under a chemical fume hood. The animals were randomly divided into the following two groups: sham unexposed (control group; *n* = 12) and exposed animals (SM group; *n* = 12). Rabbits were anesthetized via an intramuscular (i.m.) injection of a mixture of ketamine and medetomidine (15.0 mg kg^−1^; 0.25 mg kg^−1^), and then buprenorphine (0.02 mg kg^−1^) was administered subcutaneously (s.c.) to the animals. A buprenorphine delivery pump (Alzet osmotic pump, model 2 mL 1–10, Charles River Laboratories, France) was also implanted aseptically in the middle of the scapula to deliver a continuous and constant infusion (10 μL h^−1^, 7 days) of pain control medication. Animals were exposed to SM or sham-exposed in the right eye. For SM vapor exposure, 10 μL of neat SM was deposited on a paper filter placed into a 13 mm vial cap whose diameter fits with the rabbit cornea (~12 mm). An empty cap was used for the control group. Caps were then returned and fixed on the right rabbit eye using an ophthalmic gel (Ocry Gel, TVM Lab, Lempdes, France). The duration of exposure was 6 min. Eyes were then decontaminated 4 h post exposure using physiological serum. In each group, 6 animals were euthanized at day 1 (D1) and 6 at day 28 (D28) post exposure. Rabbits were firstly anesthetized using a mixture of ketamine and medetomidine (15.0 mg kg^−1^; 0.25 mg kg^−1^, i.m.) and were then euthanized via the administration of a lethal intracardiac injection of pentobarbital (333 mg kg^−1^).

#### 2.2.3. Sample Collection

Tears samples (10 µL) were collected using a calibrated Mitra micro sampling device (Trajan Scientific, Ringwood, Australia) consisting of a hydrophilic porous material. Tears were collected in both exposed and sham animals before exposure (D0, *n* = 12), just after decontamination (5 h, *n* = 12), and on D1 (*n* = 12). Other samples were collected on D3, D8, D15, and D21 post exposure (*n* = 6). For technical reasons, only 3 or 4 animals could be collected at D28. Tears were also collected before the day of treatment (D-12, D-7, D-6, D-4). In order to prevent unnecessary stress associated with repeated sample collection under conditions where biomarker formation was not expected, tears were collected only on a few randomly selected animals at each of these early time points. The data obtained in these samples were combined in a “D < 0” group (*n* = 8 in exposed and sham animals). Tear samples were frozen (−80 °C) until use. During the study, blood sampling was performed in animals sacrificed at D28 (6 SM-exposed and 6 control rabbits). For that purpose, lidocaine/prilocaine cream was applied 30 min prior to blood sampling. Blood samples were collected into lithium heparin tubes from the ear marginal vein at D-7, D1, D3, D8, D15, D21, and D28. Blood plasma was isolated using centrifugation (2000× *g*, 10 min, 4 °C) on a Heraus Megafuge 40R centrifuge (Thermo Fisher Scientific). Aqueous humor, vitreous humor, brain, liver, and lungs were collected after sacrifice. All samples were stored at −80 °C. They were then sent in dry ice for analysis with a description of collection time and exposure status.

### 2.3. Quantification of Biomarkers

#### 2.3.1. Sample Preparation

Biomarkers of sulfur mustard were quantified through ultra-high-performance liquid chromatography coupled to tandem mass spectrometry (UHPLC-MS/MS) with isotopic dilution. Analyses were performed either in tears, aqueous humor, vitreous humor and plasma, or in the nuclear DNA extracted from various organs. Sample preparation protocols were the following:

- Tears were extracted from the micro sampling device (10 μL Mitra microsamplers, Neoteryx, Trajan Scientific, Milton Keynes, UK) through incubation at 4 °C for 30 min in 200 µL of a 50:50 (*v*/*v*) mixture of methanol and water [31], together with 0.7 of a 1 µM solution of isotopically labeled internal standards. The samples were centrifuged (10 min, 10,000× *g*). The supernatants were collected and filtered in a centrifuge tube fitted with a 0.2 µM nylon membrane. Methanol was removed under vacuum, and the resulting solutions were transferred into HPLC vials. They were freeze-dried (Alpha 1-2 Christ system equipped with an Edwards E2M30 vacuum rotary pump), and the obtained residues were solubilized in 70 µL of water (final concentration of internal standards: 0.01 µM).
- Next, 100 µL of aqueous humor was mixed with 10 µL of a 0.1 µM solution of internal standards. The resulting mixture was filtered in centrifuge tubes first at 0.2 µm (10 min at 8000× *g*) and then at 10 kDa (10 min at 8000× *g*). The filtrates were transferred to HPLC vials.
- Vitreous humor is a viscous protein-rich medium. A total of 100 µL was collected with a positive displacement Microman pipette (Gilson, Middleton, WI, USA) fitted with capillary piston tips. The samples were mixed with 10 µL of a 0.1 µM solution of internal standards. Chloroform (100 µL) was added, and the samples were vigorously vortexed. They were then centrifuged, and the upper aqueous phase was collected. It was filtered in centrifuge tubes first at 0.2 µm (10 min at 8000× *g*) and then at 10 kDa (10 min at 8000× *g*). The filtrates were transferred to HPLC vials.
- Plasma samples (50 μL) were diluted in 50 μL of water and spiked with internal standards (1 μL, 10 μM). The samples were loaded onto a 0.2 μm filtration column (VWR) and centrifuged at 10,000× *g* for 5 min. The filtrate was collected and loaded onto a 30 kDa Nanosep^®^ tube. This second filtration step involved centrifugation for 20 min at 10,000× *g*. The filtrate was transferred into HPLC vials.
- Nuclear DNA was extracted from organs (brain, liver, lungs) using a previously described two-step protocol [32]. Briefly, the samples were mechanically grinded using a Potter Elvehjem homogenizer in suspension in 1.5 mL of a Triton X100 containing buffer. Nuclei were collected through centrifugation and further lysed in a sodium dodecyl sulfate (SDS)-based buffer. The samples were treated by RNase and then incubated with protease. DNA was then precipitated through the addition of a saturated solution of NaI and isopropanol. After rinsing with 40% isopropanol and 70% ethanol, the pellets were enzymatically hydrolyzed. A two-step protocol involving firstly nuclease P1, DNaseII, and phosphodiesterase II (pH 5.5, 37 °C, 2 h) and secondly phosphodiesterase I and alkaline phosphatase (pH 8, 37 °C, 2 h) was applied. Samples were then incubated for 20 min at 90 °C in a heating and mixing block (model MB-102, Bioer) to release depurinated SM guanine adducts. Samples were filtered at 30 kDa and transferred into HPLC vials

#### 2.3.2. Ultra-High-Performance Liquid Chromatography–Tandem Mass Spectrometry (UHPLC MS/MS) Analyses

UHPLC separations were performed on an ExionLC system (SCIEX) with online solid-phase extraction. The mobile phase A consisted of 5 mM ammonium formate (AmF) in MilliQ water, and mobile phase B was 5 mM AmF with 30% ACN. The samples were injected (50 μL) in a Macherey Nagel Nucleodur^®^ PFP column (50 mm × 2.0 mm ID, 5 μm particle size) used as the pre-purification column. The flow rate was set to 150 μL min^−1^, the column temperature was maintained at 50 °C, and the sample storage compartment was set to 15 °C. The initial solvent composition was 100% A. After 1 min, samples were transferred through backflush into the analytical column (EC 100/2 Nucleodur C18 HTec, 100 × 2 mm ID, 1.8 μm particle size, Macherey Nagel, Düren, Germany). The analytical gradient (flow rate 300 µL min^−1^) began with a plateau at 100% A for 1.5 min. The proportion of B linearly increased to 100% at 6 min. The latter composition was kept for 5 min before changing back to the initial conditions. DNA samples were analyzed without online SPE. The main difference with the method described above was that samples were directly injected into the analytical column. The analytical gradient was the same. Detection was performed using a 6500+ QTrap spectrometer (SCIEX) operated in the “multiple reaction monitoring mode” (MRM). For each analyte and corresponding internal standard, an MRM transition was used for quantification, together with an additional qualifying signal (Table 1). 

## 3. Statistical Analysis

Statistical analyses were performed with GraphPad Prism 11.0.1 software (Boston, MA, USA). Data were collected in both the right and left eyes in both exposed and sham animals. For each type of sample, the number of exposed and sham animals was the same. Kruskal–Wallis tests followed by Dunn’s multiple comparisons were used to analyze data from time course studies in aqueous and vitreous humors (*n* = 6). Results in tears were paired values, and their statistical analysis was based on repeated-measures ANOVA followed by Dunnett’s multiple comparison test. Because animals were sacrificed at D1, analyses were performed separately for short times (D0, 5 h, D1: *n* = 12) and longer times (D3, D8, Q15, and D21: *n* = 6 in each group). In order to optimize the number of animals with a complete set of data, results in the D < 0 and D28 groups were not included. Additional statistical analysis of data at 5 h was based on either a Wilcoxon test (paired values for right and left eyes of the same animals) or a Kolmogorov–Smirnov test (between exposed and sham animals). Results were considered significant with *p* ≤ 0.05.

## 4. Results

The preparation protocols designed for each type of sample were found to be suitable for the sensitive analysis of SM biomarkers. This was shown by large recovery (>90%) of the isotopically labeled internal standards. The NAC-SM conjugate was detected in none of the samples, although its detection was more sensitive than that of Cys-SM (limit of detection of 12 and 83 fmol for NAC-SM and Cys-SM, respectively). The limit of detection of Gua-SM was slightly lower (2 fmol) than that of NAC-SM. Tiny interfering peaks were present in most of the chromatograms of biological samples but not of standards.

## 5. Biomarkers in Tears

A large number of samples was available for tears. Samples were collected in both control and exposed groups from as early as 12 days before exposure and up to 28 days after. In addition, samples were collected from both the right eye, which was actually exposed to SM, and the left eye, which served as an internal control for each animal. Cys-SM was unambiguously detected in the exposed eyes in the hours following contamination as well as at D1 after decontamination (Figure 1a). Cys-SM was not detected in the left eye, which remained unexposed (Figure 1b). The concentration of Cys-SM in the right and left eyes of sham unexposed animals was also at the limit of detection (data available at https://zenodo.org/records/23180591, accessed on 13 October 2026). Similar results were obtained for the Gua-SM biomarker. No significant amount was detected above background in the eyes of the sham unexposed animals or before exposure in the exposed group. A significant increase in the concentration was observed 5 h and 1 day after SM exposure in the right eye (Figure 2a). This effect was below significance after 3 and 8 days. No Gua-SM was detected either in the left eye of exposed animals, as illustrated at 5 h (Figure 2b), or in the eyes of the controls (data at 5 h in Figure 2b, other values available at https://zenodo.org/records/23180591, accessed on 13 October 2026).

## 6. Biomarkers in Aqueous and Vitreous Humors

We then quantified the level of biomarkers in aqueous and vitreous humors collected in animals euthanatized on D1 or D28 after exposure. In both types of samples, the level of Cys-SM and Gua-SM was significantly higher at D1 in the exposed right eye compared to the left eye, which serves as an internal control, and to the right eye of control animals (Table 2). In exposed eyes at D1, the concentration in biomarkers was one order of magnitude higher in aqueous compared to vitreous humor. At D28, the concentration of biomarkers in the SM exposed right eye was as low as in the control left eye or in the right eye of the sham exposed animals (Table 2).

## 7. Biomarkers in Plasma and Internal Organs

Diffusion of SM outside of the ocular sphere was first evaluated by the ability to detect SM adducts in blood plasma. The concentration in all samples was below the limit of detection at all time points whether rabbits were exposed (Figure 3) or not. Potential diffusion of SM to other organs was also investigated by quantifying Gua-SM in nuclear DNA. Although the limit of detection of the assay was below 2 fmol, the SM adduct was not detected in any of the three investigated organs: brain, lungs, or liver (Figure 4). Likewise, NAC-SM was not detected (data not shown).

## 8. Discussion

The purpose of the present investigation was to characterize the extent of damage induced by SM following ocular exposure both in terms of localization and kinetics. This work, carried out in rabbits, relied on the quantification of a series of molecular biomarkers. The protocol involved a uniform vapor corneal exposure without lateral diffusion. This should be kept in mind when extrapolating the results to actual exposures that may combine liquid or mixed vapor/liquid mixtures. We first focused on the eyes, with emphasis placed on three different compartments: the tears, the aqueous humor, and the vitreous humor. Tears are easily accessible media that have been used for biomarker quantification in several pathologies [33,34,35,36,37,38,39]. The impact of SM exposure on tears has been investigated, but only in terms of proteome modulation [40,41]. Aqueous humor is another investigated medium for the search of biomarkers in a wide range of pathologies [42,43,44,45], while vitreous humor has been less frequently studied [46,47,48], likely because of its more limited accessibility. Only biochemical and inflammatory markers in the aqueous humor have been described after SM ocular exposure before and during the onset of clinical signs [49]. To the best of our knowledge, no data on the impact of SM is available on vitreous humor.

A first interesting observation provided by our UHPLC-MS/MS assay was the lack of *N*-acetylcysteine conjugate in tears and aqueous and vitreous humor. Acetylation of the cysteine adducts is the final step of metabolism of glutathione conjugates in the mercapturic pathway. It is expected to occur predominantly in kidneys and, to a lesser extent, in some other organs [50,51,52]. Accordingly, we detected NAC adducts in the blood plasma and the brain of mice following cutaneous exposure to CEES [53]. NAC-SM was also detected in the urine of topically SM-exposed rats [54,55]. The absence of NAC adducts in our samples could potentially be explained by specificities in their metabolism. First, very efficient clearance of NAC-SM could take place. However, in this case, the NAC adduct would be detected in surrounding biological fluids, such as tears. In addition, active clearance of *N*-acetylated cysteine metabolites in the eye has, to our knowledge, never been reported. A second possible metabolic explanation to the lack of NAC-SM could be limited formation of its cystein substate. However, we unambiguously detected Cys-SM in all exposed samples, indicating availability of the corresponding precursor. Our results thus suggest that the studied ocular compartments exhibit only very low *N*-acetyl-transferase enzymatic activities. This hypothesis is in agreement with available data showing that, in the eyes, *N*-acetyl-transferase is mostly expressed in the cornea and much less in the lens [56,57]. An additional explanation could be the occurrence of methyl-thiolation in the lens, as reported for the metabolism of naphthalene [58].

In contrast, the two other investigated biomarkers, Cys-SM and Gua-SM, were unambiguously detected in the exposed eyes. Non-invasive measurements in tears showed that both SM adducts were present at the earliest investigated time point after exposure (5 h) and remained detectable 1 day after decontamination. The concentrations returned to background levels after 8 days. The rapid formation of the biomarkers followed by a return to baseline was also observed in aqueous and vitreous humors analyzed after sacrifice at D1 and D28. The concentration of Cys-SM and Gua-SM was found to be lower in tears than in aqueous humor. This can be explained by the continuous turnover of tears in the eyes, which are constantly renewed and cleared from the ocular surface. In contrast, aqueous humor is located behind the cornea, which is the most exposed part of the eye.

Our quantitative analysis of SM biomarkers revealed a distinct decreasing concentration gradient across the ocular compartments, with vitreous humor exhibiting the lowest concentration in biomarkers. This observation is in line with the observation of 20-times lower binding of ^14^C-labeled SM in vitreous compared to aqueous humor [59]. It should nevertheless be stressed that the concentration in biomarkers may not be directly proportional to the SM concentration in the different compartments, as it also depends on the availability of the targets and enzymatic activities. For example, glutathione-*S*-transferase activity is not homogeneous throughout the eye and is more expressed in the retina than in other ocular compartments [60]. Glutathione itself is present in different concentrations in ocular compartments in the decreasing order of lens > vitreous humor > aqueous humor [61]. However, the fact that similar trends were observed for two biomarkers of very different origin (GSH and nucleic acids) suggests that differences in metabolism and substrate availability are unlikely to have a major impact on the results. This anteroposterior gradient provides valuable insight into the toxicokinetic and physical barriers governing SM intraocular diffusion. The maximal concentration of biomarkers in the tear film and aqueous humor reflects the immediate dissolution of the agent upon initial contact with the ocular surface. As detailed by McNutt et al. [62], SM is highly lipophilic and rapidly penetrates the biological membranes of the corneal epithelium. Then, SM is rapidly involved in irreversible alkylation reactions of local macromolecular targets, including DNA and proteins. This rapid chemical consumption acts as a “metabolic and structural sink”, drastically reducing the amount of unreacted, free toxicant capable of migrating deeper into the eye. The significant drop in biomarker levels observed between the aqueous and vitreous humors further emphasizes the role of internal anatomical barriers. The crystalline lens, along with its dense capsule, serves as a first physical shield that compartmentalizes the eye. This structural compartmentalization is further reinforced by the physiological dynamics of the aqueous humor because the aqueous fluid is continuously produced by the ciliary body and drained anteriorly through the trabecular meshwork. Consequently, while SM can diffuse past the corneal stroma to inflict endothelial and anterior uveal damage, the combination of rapid tissue alkylation and the lens barrier successfully insulate the vitreous humor and the posterior pole from significant direct toxic exposure. Accordingly, the cornea was reported to be by far the ocular tissue accumulating the largest fraction of ^14^C-labeled SM after exposure in the eye [59].

The present results show that SM is persistent enough in exposed eyes to react with biomolecules. We thus investigated whether SM could also diffuse out of the ocular sphere and reach other organs. This process was found to be very efficient in skin, as is well-documented [63,64]. Accordingly, dermal SM exposure of mice induced the formation of DNA adducts not only in cutaneous cells but also in the brain, lungs, kidney, and spleen [65,66]. A similar experiment with CEES resulted in the formation of adducts in brain DNA [53]. Consequently, we searched for the presence of DNA adducts in the DNA of the brain, lungs, and liver of ocularly exposed rabbits but could not detect them. Similarly, we did not detect NAC-SM, Cys-SM, or Gua-SM adducts in blood plasma. These results contrast with reports of the presence of molecular biomarkers in urine following dermal exposure of rats to SM [54,66,67] and mice to CEES [53]. The fact that we did not observe biomarkers in the plasma of rabbits is more evidence of different behavior of SM after either cutaneous or ocular contamination. A last observation ruling out massive diffusion of SM from the ocular sphere is that exposure of the rabbits in the right eye did not lead to the formation of biomarkers in the left eye in spite of their close proximity. The bulk of these observations are in line with the role of the cornea as a major barrier against the diffusion of SM. Interestingly, we recently studied the impact of rabbit ocular exposure to SM on their metabolomic plasma profiles using the same protocol as that used in the present study. The metabolomic changes observed in the plasma were mainly associated with the systemic impact of the ocular tissue injury (oxidative stress, vascularization) and the associated inflammation. The collected data did not support a major role played by the diffusion of SM from the eyes to other internal targets [27]. It may thus be proposed that the biomarkers detected in patients exposed to SM [29,68,69,70,71,72] may predominantly originate from systemic diffusion through the skin and lungs rather than from the eyes.

## 9. Conclusions

The analytical developments performed in the present work allowed us to show that ocular exposure led to the formation of SM adducts to biomolecules in the eyes. The analysis of multiple biological compartments showed the existence of an anteroposterior gradient of biomarkers, while the analysis of plasma and internal organs failed to provide evidence of systemic diffusion, in contrast to what has been reported following cutaneous exposure. These results are consistent with the known role of internal anatomical barriers played by the crystalline lens and aqueous and vitreous humors in limiting the passive diffusion of toxicants from the anterior chamber to the posterior segment. Further characterization of the ocular tissue injury will be performed and compared to the extent and time dependance formation of the studied biomarkers’ formation. These pieces of information will be of major importance for the development of simple detection tools that can be easily transported and used in the field. In particular, detecting sulfur mustard adducts in tears could serve as a readily accessible and non-invasive test for the rapid identification and triage of exposed individuals following a sulfur mustard incident.

## Figures and Tables

**Figure 1:**
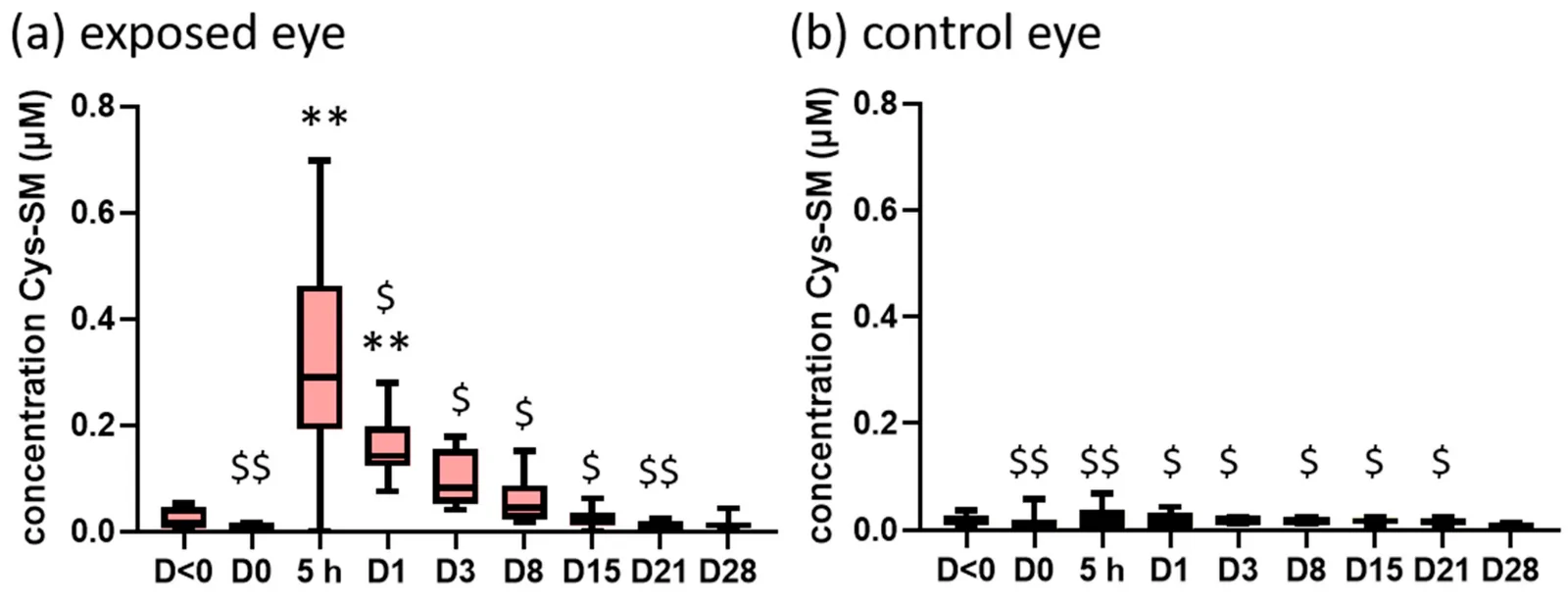
Time course variation of the concentration of Cys-SM in tears collected from the eyes of rabbits: (**a**) right eye exposed to SM; (**b**) unexposed left eye. The reported data are the median, the first and third quartiles, and the minimum and maximum values. Statistical analysis of the paired values was based on repeated-measures ANOVA followed by Dunnett’s multiple comparison test (D0, 5 h, D1: *n* = 12 and D3, D8, Q15, D21: *n* = 6). Significance of the differences was tested with respect to the values measured in SM exposed right eye at D0 (** < 0.01) or at 5 h ($: *p* < 0.05, $$ < 0.01).

**Figure 2:**
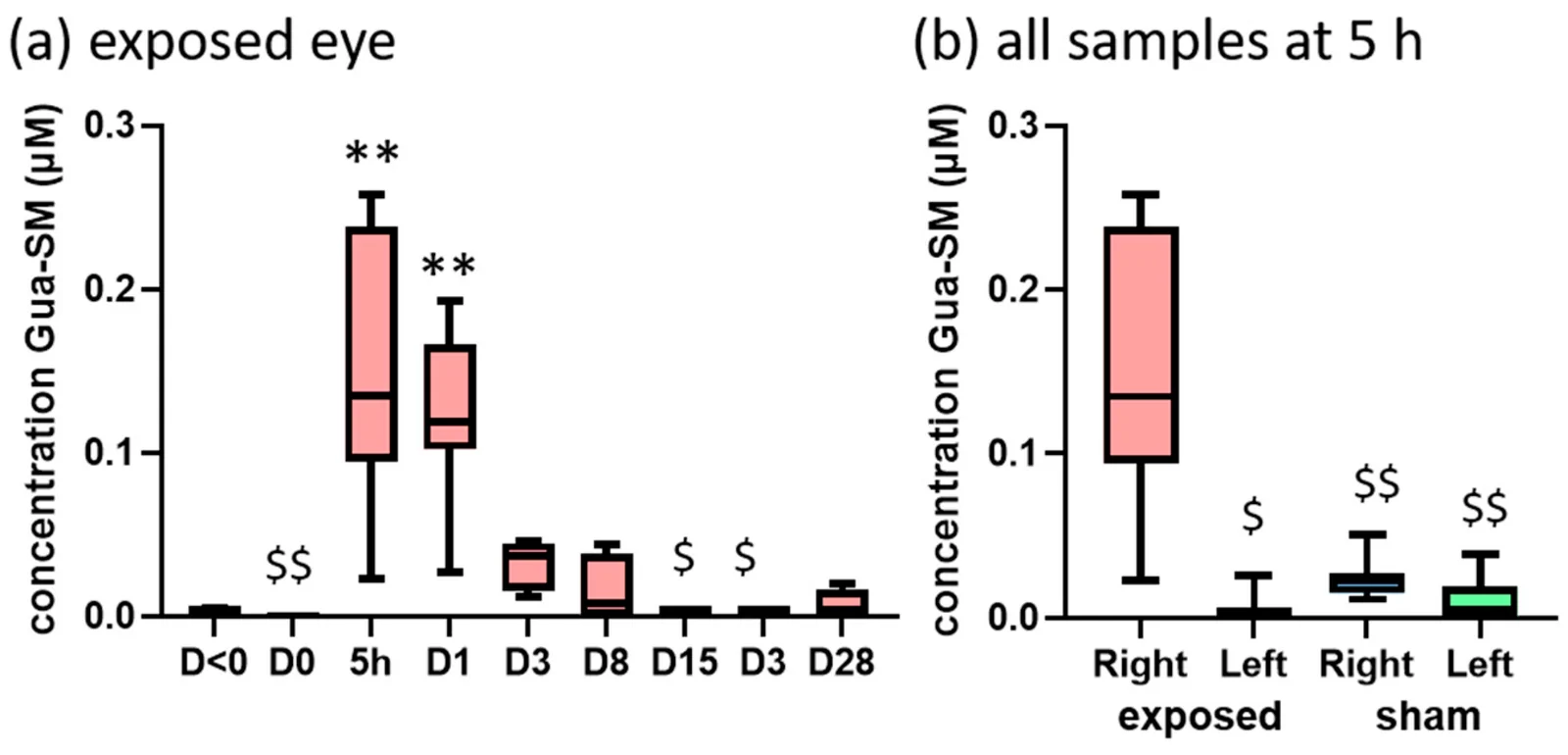
Concentration of Gua-SM in tears collected (**a**) at different time points in the right eyes (exposed) of rabbits up to 28 days after exposure to SM and (**b**) 5 h post exposure in the right (exposed) and left (unexposed) eyes of exposed or Sham animals. The reported data are median, the first and third quartiles, and the minimum and maximum values. Statistical analysis of the paired values (**a**) was based on repeated-measures ANOVA followed by Dunnett’s multiple comparison test (D0, 5 h, D1: *n* = 12, and D3, D8, Q15, D21: *n* = 6). Statistical analysis of data at 5 h (**b**) was based on either a Wilcoxon test (paired values for right and left eyes) or a Kolmogorov–Smirnov test (between exposed and sham animals). Significance of the differences was tested with respect to the value measured in SM exposed right eye at D0 (** < 0.01) or at 5 h ($: *p* < 0.05, $$ < 0.01).

**Figure 3:**
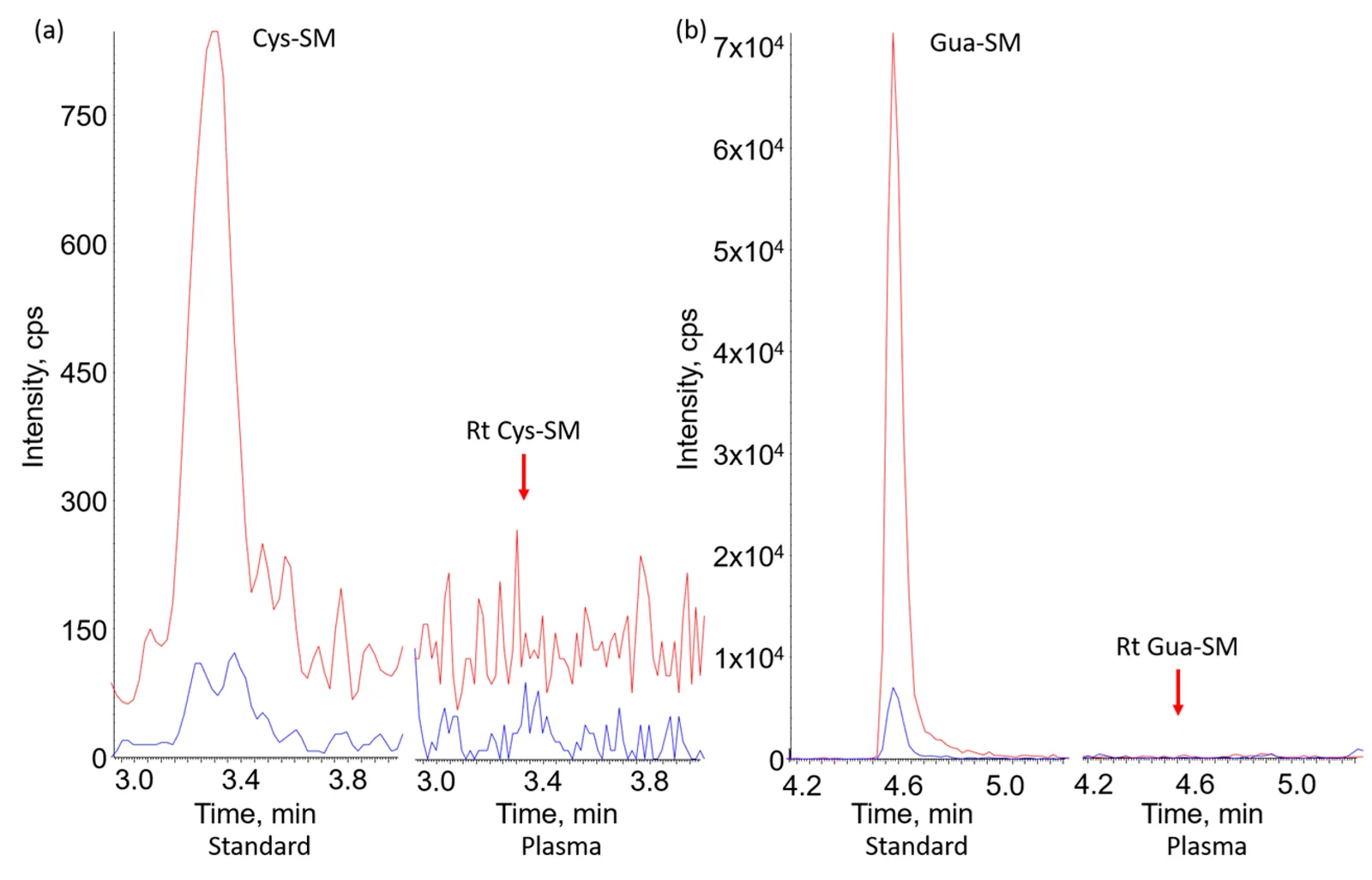
Detection of (**a**) Cys-SM and (**b**) Gua-SM in blood plasma. The reported data are typical chromatograms obtained upon UHPLC-MS/MS analysis of either a solution of authentic standards or a plasma sample of an exposed rabbit at D1 after SM ocular exposure. The red arrows indicate the retention time (Rt) of the biomarker. The different traces correspond to the monitored fragmentations in the MRM detection mode listed in Table 1.

**Figure 4:**
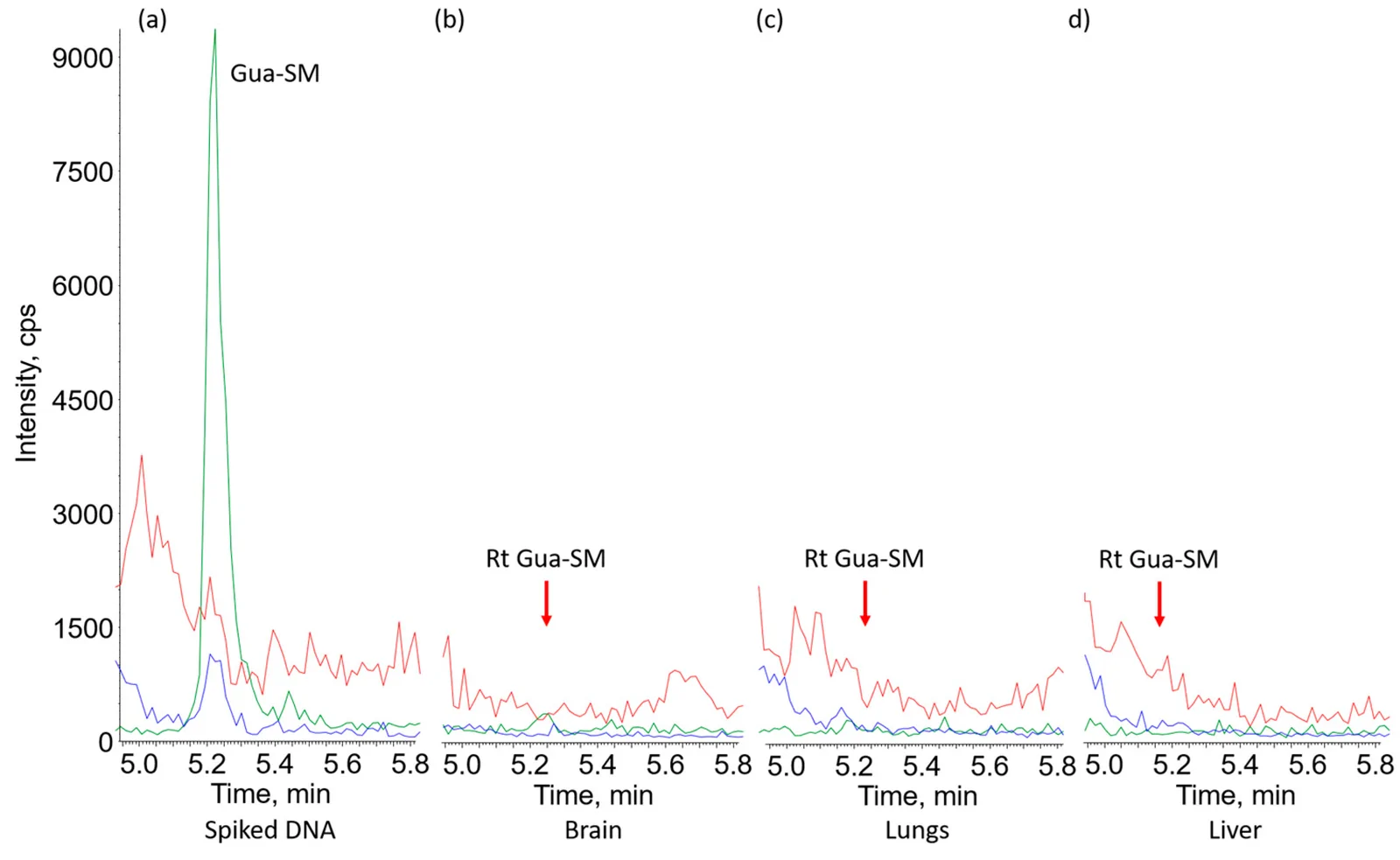
Detection of Gua-SM in nuclear DNA of internal organs at D1 after SM ocular exposure of a rabbit. The reported chromatograms correspond to (**a**) DNA spiked with 7 femtomoles of authentic Gua-SM, (**b**) brain DNA, (**c**) liver DNA, and (**d**) lung DNA. The red arrows indicate the retention time (Rt) of Gua-SM. The different traces correspond to the monitored fragmentations in the MRM detection mode listed in Table 1.

**Table 1:** Parameters applied to the multiple reaction monitoring (MRM) detection of SM biomarkers. Abbreviations and units: Q1 and Q3: *m*/*z* of parent and product ions; DP: declustering potential (V); EP: entrance potential (V); CE: collision energy (V); CXP: collision cell exit potential (V).

Targeted Analyte	Q1 (*m*/*z*)	Q3 (*m*/*z*)	DP (V)	EP (V)	CE (V)	CXP (V)
Cys-SM	226.2	61	20	5	40	10
		105	50	5	20	15
[M + 4]-Cys-SM	230.2	61	20	5	40	10
		105	50	5	20	15
Gua-SM	256.2	61	20	5	40	30
		105	20	10	20	30
[M + 5]-Gua-SM	261.2	61	20	5	40	30
		105	20	10	20	30
NAC-SM	268.3	84	50	10	40	10
		162	20	10	20	20
[M + 4]-NAC-SM	272.3	87	50	10	40	10
		166	20	10	20	20

**Table 2:** Concentration (expressed in µM) of Cys-SM and Gua-SM in the aqueous and vitreous humors of rabbits exposed to SM *.

Biomarker	Exposed D1 Right	Exposed D28 Right	Exposed D1 Left	Exposed 28 Left	Sham D1 Right	Sham 28 Right
Aqueous humor						
Cys-SM	20.7 ± 11.5	0.1 ± 0.5 $	0.2 ± 0.2 $	0.0 ± 0.0 $$	0.1 ± 0.0 $	0.2 ± 0.1 $
Gua-SM	3.4 ± 1.0	0.1 ± 0.0 $	0.1 ± 0.0 $	0.1 ± 0.1 $$	0.0 ± 0.0 $$	0.1 ± 0.0 $
Vitreous humor						
Cys-SM	2.2 ± 1.2	0.2 ± 0.3 $	0.0 ± 0.0 $	0.0 ± 0.1 $	0.0 ± 1.1	0.1± 0.2 $
Gua-SM	0.11 ± 0.05	0.01 ± 0.01 $	0.01 ± 0.00 $	0.01 ± 0.01 $	0.01 ± 0.01 $	0.01 ± 0.01 $

*: The right eye was exposed, and the left eye served as a control. The reported data are the median and the difference between first and third quartiles. The reported zero values result from the rounding of low numbers. For each marker in each organ, the concentration measured in all sample was compared to that in the exposed right eye at D1. Statistical analysis was based on a Kruskal–Wallis test, followed by Dunn’s multiple comparison test (*n* = 6, $: *p* < 0.05, $$ < 0.01).

## Data Availability

The original data on tears, aqueous humor, and vitreous humor presented in this study are openly available in the Zenodo repository at https://zenodo.org/records/23180591 (accessed on 6 October 2026). All of the MRM chromatograms showing the absence of biomarkers in plasma and internal organs are available upon request from the corresponding author.

## Abbreviations

The following abbreviations are used in this manuscript:

SM	Sulfur mustard
CEES	2-chloroethyl-ethyl-sulfide
NAC-SM	Sulfur-mustard adduct to *N*-acetylcysteine
Cys-SM	Sulfur-mustard adduct to cysteine
Gua-SM	Sulfur-mustard adduct to guanine
AmF	Ammonium formate
UHPLC-MS/MS	Ultra-high-performance liquid chromatography coupled to tandem mass spectrometry
MRM	Multiple Reaction Monitoring detection mode
